# Emerging Role of Hydrogels in Drug Delivery Systems, Tissue Engineering and Wound Management

**DOI:** 10.3390/pharmaceutics13030357

**Published:** 2021-03-08

**Authors:** Shery Jacob, Anroop B. Nair, Jigar Shah, Nagaraja Sreeharsha, Sumeet Gupta, Pottathil Shinu

**Affiliations:** 1 Department of Pharmaceutical Sciences, College of Pharmacy, Gulf Medical University, Ajman 4184, United Arab Emirates; 2 Department of Pharmaceutical Sciences, College of Clinical Pharmacy, King Faisal University, Al-Ahsa 31982, Saudi Arabia; anair@kfu.edu.sa (A.B.N.); sharsha@kfu.edu.sa (N.S.); 3 Department of Pharmaceutics, Institute of Pharmacy, Nirma University, Ahmedabad 382481, India; jigsh12@gmail.com; 4 Department of Pharmaceutics, Vidya Siri College of Pharmacy, Off Sarjapura Road, Bangalore 560035, India; 5 Department of Pharmacology, M. M. College of Pharmacy, Maharishi Markandeshwar (Deemed to Be University), Mullana 133203, India; sumeetgupta25@gmail.com; 6 Department of Biomedical Sciences, College of Clinical Pharmacy, King Faisal University, Al-Ahsa 31982, Saudi Arabia; spottathail@kfu.edu.sa

**Keywords:** hydrogel, stimuli responsive, polymeric hydrogel nanoparticles, drug delivery systems, wound dressing materials, tissue engineering scaffolds, modified contact lens

## Abstract

The popularity of hydrogels as biomaterials lies in their tunable physical properties, ability to encapsulate small molecules and macromolecular drugs, water holding capacity, flexibility, and controllable degradability. Functionalization strategies to overcome the deficiencies of conventional hydrogels and expand the role of advanced hydrogels such as DNA hydrogels are extensively discussed in this review. Different types of cross-linking techniques, materials utilized, procedures, advantages, and disadvantages covering hydrogels are tabulated. The application of hydrogels, particularly in buccal, oral, vaginal, and transdermal drug delivery systems, are described. The review also focuses on composite hydrogels with enhanced properties that are being developed to meet the diverse demand of wound dressing materials. The unique advantages of hydrogel nanoparticles in targeted and intracellular delivery of various therapeutic agents are explained. Furthermore, different types of hydrogel-based materials utilized for tissue engineering applications and fabrication of contact lens are discussed. The article also provides an overview of selected examples of commercial products launched particularly in the area of oral and ocular drug delivery systems and wound dressing materials. Hydrogels can be prepared with a wide variety of properties, achieving biostable, bioresorbable, and biodegradable polymer matrices, whose mechanical properties and degree of swelling are tailored with a specific application. These unique features give them a promising future in the fields of drug delivery systems and applied biomedicine.

## 1. Introduction

Hydrogels are hydrophilic polymers composed of three-dimensional viscoelastic networks that retain water many times their dry weight and swell in physiological environments. The physical interactions and chemical cross-linking of hydrogels can contribute towards both structural and physical integrity [1]. The applicability of hydrogels as biomaterials lies in the uniqueness of their properties such as water content, soft and elastic consistency, and low adhesive force with water or biological fluids [2]. Due to distinct physical characteristics, hydrogels can provide controlled dissolution, protect the labile drug from degradation and control the release of various actives, including small-molecule drugs, macromolecules, and cells. Thus, hydrogels have the unique ability to solve many formulation- and drug-related issues, thereby making them suitable for utilization in various drug delivery systems, wound dressings, hygiene products, and regenerative medicine. Apart from other published literature, the present article provides a comprehensive overview on the recent advances of hydrogels in diverse drug delivery systems. Furthermore, the applicability of hydrogels in wound management and tissue engineering are extensively covered.

Hydrogels can be broadly classified based on the characteristics of side groups (ionic or non-ionic), structural aspects (homo or copolymer), physical nature (crystalline, amorphous, supramolecular), and responsiveness to various external stimuli such as temperature, pH, light, ionic strength, ultrasound, electromagnetic radiation, glucose, and proteins. In order to impart adequate mechanical strength to hydrogels besides biodegradability, various types of physical and chemical cross-linking methods are currently being employed. A comprehensive overview on the synthesis of hydrogels by physical cross-linking and chemical cross-linking techniques, materials, procedures, advantages, and disadvantages are depicted in Table 1. The next section discusses the classification of hydrogels based on their responsiveness to various external stimuli. Different types of stimuli-responsive hydrogels are summarized in Table 2.

## 2. Stimuli-Sensitive Hydrogels

### 2.1. Thermoresponsive

Due to their ability to undergo large phase transitions triggered by temperature, thermoresponsive hydrogels can incorporate drugs that can be slowly released from the interior of the gel. A synthetic temperature-sensitive graft copolymer based on *N*-isopropylacrylamide (NIPAAm) demonstrated excellent chemical stability, hydrophilicity, adequate swelling, and minimum lower critical solution temperature suitable for drug delivery systems [20]. The sol-gel transition temperature of thermosensitive hydrogel shifts to a higher temperature by increasing the butyl methacrylate content [21]. They are favorably exploited for the delivery of macromolecular drugs. Various biomedical applications of thermoresponsive hydrogels are described in another review [22].

### 2.2. pH Sensitive

This class of hydrogels exhibits pH-dependent swelling because of the changes in the hydrophobic/hydrophilic characteristics of the polymer chain and non-covalent interactions. The extent of swelling is influenced by pH, dissociation constant, and degree of dissociation of charged groups, polymer level, and pH of the external medium. Unlike temperature changes, variation in pH exists in the different regions of the body and in certain disease conditions such as chronic wounds and tumors; this property can therefore be utilized for targeted drug delivery to cellular compartment or particular tissues [23].

Amphiphilic pH-sensitive hydrogels prepared in different molar ratios of hydrophilic poly(methacrylic acid–grafted–ethylene glycol) conjugated with hydrophobic poly (methyl methacrylate) (PMMA) nanoparticles have been reported [24]. The oral administration of a polymeric carrier subsequently resulted in the release of entrapped drugs to the intestine triggered by a shift in pH. The study indicated that a combination of pH-responsive nanoparticles with biocompatible hydrogels could deliver the drugs to the various sites of the gastrointestinal tract. The pH-sensitive hydrogels have a significant role in the fabrication of biosensor, biomedical, and drug delivery applications [25].

Smart hydrogels such as dual sensitive pentablock copolymers were synthesized through chemical conjugation between thermosensitive segment, poly(ε-caprolactone)-*b*-poly (ethylene glycol)-*b*-poly(ε-caprolactone) (PCL-*b*-PEG-bPCL), and pH-responsive polyamide [26]. The pentablock copolymers showed gelation at biological environment (pH 7.4, 37 °C), a controlled degradation rate and hence controlled release of the entrapped compound. Chitosan nanoparticles loaded with insulin were included and 15wt%–35wt% pentablock copolymer-controlled release of the agent was noticed without any initial burst [27].

### 2.3. Photoresponsive

Photoresponsive hydrogels generally consist of a photoreactive chromophore as the functional part of the polymer chain. A photoresponsive supramolecular hydrogel constituted of cyclodextrin or azobenzene modified dextran was investigated as a controlled release delivery system of proteins [28]. Light-responsive hydrogel could be a potential delivery platform in different areas such as gene delivery and tissue engineering [29]. A novel infrared-responsive poly (*N*-isopropylacrylamide) (PNIPAAm) hydrogel nanocomposite incorporating glycidyl methacrylate functionalized graphene oxide (GO–GMA) demonstrated large water uptake and thus has the potential to be employed in microfluidic devices [30].

### 2.4. Electroresponsive

Polyelectrolytes under an applied electric field may either swell, contract, or bend and this property has facilitated its utilization in the drug delivery field, tissue engineering, or biomimetic systems. An electroresponsive hybrid hydrogel prepared from multi-layered carbon nanotube/poly (methylacrylic acid) has been studied [31]. In vitro and in vivo studies displayed a pulsatile drug release profile after an applied electric field. Biocompatible electroresponsive hydrogels composed of polyacrylic acid and fibrin were found to stimulate cell growth and tissue formation [32].

### 2.5. Ultrasonically Responsive

Ultrasound is widely used in biomedical applications due to its non-invasive nature, safety, relatively high precision, and capacity to penetrate tissues [33]. Ultrasound polymer systems can be guided by lasers and MRI systems so as to control drug delivery rates suitable for specific disease or condition. It has been demonstrated the ultrasonically controlled release of ciprofloxacin from self-assembled coatings on poly (2-hydroxyethyl) methacrylate hydrogel significantly reduced the biofilm accumulation.

### 2.6. Magnetoresponsive

A polymeric network comprises magnetic micro/nanoparticles which can be maneuvered under an external magnetic field so that an effective concentration can be maintained at the target site while minimizing side effects [34]. Magnetically stimulated hydrogels have been evaluated for diverse drug delivery applications [35]. The drug delivery and porosity of the magnetic gel can be regulated through switching on/off the external magnetic field. Magneto-responsive hydrogels have been fabricated to improve the scaffold performance such as restoring, maintaining, and improving tissue functions, and to stimulate cellular responses after an applied external magnetic field [36].

### 2.7. Enzyme Responsive

Enzyme-responsive polymers can be utilized as indicators for observing various metabolic changes and act as signals for the targeted drug delivery to various organs. An ideal self-modulated insulin drug delivery system requires glucose-sensing ability and an automatic shut-off mechanism. The biocatalytic transformations of glucose oxidase included within the pH-responsive DNA containing hydrogels provides a glucose-triggered matrix for the controlled release of insulin and therefore behaves as an artificial pancreas [37]. The cationic natural polymer oligochitosan and Ca^2+^ were allowed to cross-link with pectin to form hydrogel microcarriers to deliver the drug slowly in the upper gastrointestinal tract and rapid release of drug simulated physiological conditions of the colon [38]. The in situ triggered gel formation and disintegration of polymeric hydrogel provides them with applicability in different areas such as tissue engineering, injectable hydrogels, and controlled release delivery systems.

### 2.8. Ionic Strength Responsive

The responsiveness towards ionic strength is typically observed in polymers with ionizable groups such as polyelectrolytes. Depending on the magnitude of the electric field, they may swell, deswell, or erode and provide pulsatile drug release. It was reported that drugs with oppositely charged interpolyelectrolyte complexes acted as drug reservoirs and exhibited sustained drug release rates in an aqueous medium followed by increased drug release in physiological pH, enhanced by ionic exchange [39]. Interpolyelectrolyte complexes developed as multiparticulate systems loaded with benznidazole displayed a multi-kinetic in vitro release profile. Preclinical studies in a murine model of Chagas disease indicated an improved performance compared to conventional treatment [40]. The self-assembly of polypeptides into biocompatible and biodegradable polymersomes can also be regulated for biomedical applications with different ionic strengths [41].

### 2.9. Inflammation Responsive

The inflammatory process is mediated by B and T lymphocytes but is sustained, amplified, and mediated by polymorphonuclear leukocytes and macrophages. A naringin-carrying amphipathic carboxymethyl-hexanoyl chitosan glycerol colloidal pH-responsive hydrogel with inflammation-responsive characteristics was demonstrated in experimentally induced periodontics [42]. The pH changes at the inflammatory site resulted in drug release from the matrices and subsequent reduction of periodontal bone loss, inflammatory infiltration, and downregulated toll-like receptor, the receptor for advanced glycation end products, and tumor necrosis factor.

## 3. Functionalization Strategies of Hydrogels

The substitution with active cross-linking sites in place of covalent cross-linking points has been evaluated to decrease the damage caused by non-uniform distribution of covalent bonds. Further, these novel molecular structures distribute stress more uniformly by positional adjustment along a threaded polymer network after an applied external force. An example depicting this method is a slide-ring hydrogel prepared by a one-pot approach based on thiol-ene click chemistry [43].

Cross-linked polymer structures can be additionally reinforced by secondary interpenetrating polymer networks (IPN) (double network hydrogels). In the case of a fully and semi-interpenetrating polymer network, a second polymer is cross-linked or physically associated with the already cross-linked polymer. Under an external stress, the first network absorbs energy and a loosely cross-linked secondary polymer maintains the integrity of these double-network hydrogels. Semi-IPN chitosan-based hydrogels prepared by selective cross-linking with polyelectrolytes caused enhancement in tensile strength but a reduction in the swelling capability of the gels. These hydrogel systems were demonstrated to be responsive to different pH and ionic strength, and hence had potential application for controlled release of drugs. Many semi-IPN-based hydrogels constituted of alginate and methacrylate polymers exhibited increased porosity, multiresponsiveness, sustained release, and electrical sensitivity [44]. Ionic groups present in IPN hydrogels comprised of alginate/poly (isopropylacrylamide) are stabilized by covalent bonds as well as through ionic interactions. These polyionic complexes, having improved mechanical property, ionic, and pH responsiveness, could be used as biomaterials [45].

Nanoparticles can act as a multifunctional cross-linking point in three-dimensional polymeric structures either by physical or chemical cross-linking. Physical adsorption exists between the nanoparticles, and molecular networks with high surface areas associated with nanoparticles can enhance the mechanical strength of these nanocomposite or hybrid hydrogels [46]. Frequently reported nanomaterials in nanocomposite gels are raw material particles, polymeric, inorganic/ceramic, metal, or metal oxide nanoparticles [47]. In situ formation of zinc oxide nanoparticles within the nanocomposite carboxymethyl cellulose/zinc oxide hydrogels have been reported. The novel nanogel displayed pH- and ion-sensitive behavior with enhanced swelling in aqueous medium compared to plain hydrogel [48]. In a recent study, photocross-linked methacrylated glycol chitosan-montmorillonite nanohydrogels demonstrated tremendous increase in mechanical strength, in vitro mesenchymal cell growth, multiplication, and differentiation [49].

Presently, research is also progressing in the field of nanocomposite hydrogels, including functionalized nanomaterials [50]. The nanomaterial functionalization can promote cell–scaffold interactions, increase cross-linking between polymer and nanoparticles, and advance the self-healing capability or drug delivery. The chiral biomolecules such as proteins have been used to functionalize nanomaterials, which requires more attention due to the significant effect on cell activities. Utilizing enantiomers of biodegradable poly-d (l)-lysine (PDL and PLL) to the functionalized external surface of zeolites and periodic mesoporous organosilicas demonstrated higher affinity and migration of cells to the enantiomorph portion of the Janus nanocomposite hydrogel, preferred by the cells [51].

As a robust nanomaterial having excellent biocompatibility, biodegradability, and high mechanical strength, nanoscaled cellulose has possible applications in the areas of pharmaceutical technology and bioengineering. A self-healing cellulose nanocomposite hydrogel prepared from acylhydrazineterminated polyethylene glycol (PEG) cross-linked with dialdehyde cellulose nanocrystals showed significant increase in the mechanical strength, self-healing efficiency, and biocompatibility of the hydrogel [52]. Conductive hydrogel-based polymers and nanoparticles have unique properties, like regular hydrogels, with an additional advantage of electrical conductivity. Different synthetic routes such as single component stable conjugative conductive hydrogel and multicomponent conductive hydrogel prepared through electrochemical polymerization and chemical oxidation polymerization have been explored. Many conductive polymers such as polypyrrole, polythiophene, poly (3,4-ethylene dioxythiophene), and polyaniline are extensively employed in tissue engineering to promote cell growth and proliferation [53].

**Figure 1:**
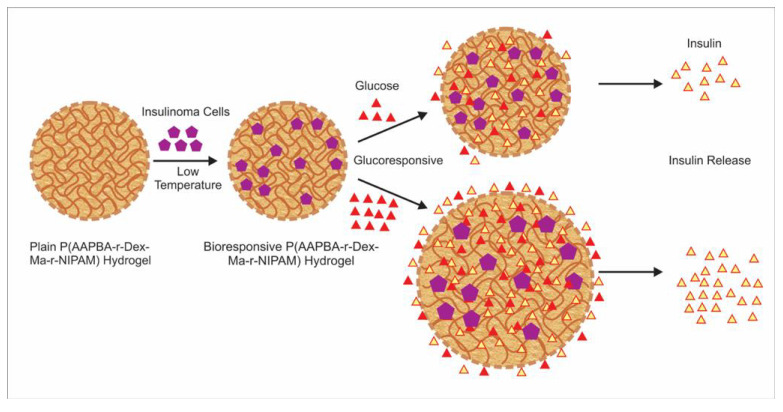
Schematic representation of the synthesis of poly(*N*-isopropylacrylamide-*co*-dextran-maleic acid-*co*-3-acrylamidophenylboronic acid) hydrogel, insulin-secreting cell encapsulation, and insulin release under glucose conditions.

Injectable hydrogels are distinguished by inherent fluidly and therefore can be administered through an injection. Depending on the methods employed, injectable hydrogels are classified into light irradiated (UV or visible) covalent bonded hydrogels and spontaneously formed self-assembling hydrogels. An injectable hydrogel with tailored porosity, improved mechanical properties, enhanced water absorbency, and diffusivity was prepared based on emulsion technique [54]. A novel injectable hydrogel made of poly(*N*-isopropylacrylamide-*co*-dextran-maleic acid-*co*-3-acrylamidophenylboronic acid) (P(AAPBA-Dex-NIPAM)), with glucose-responsiveness and thermo-responsiveness for diabetic therapy, has been developed [55]. The bioinspired hydrogel encapsulated with insulinoma cells demonstrated real-time glycemic regulation similar to pancreatic islet β cells (Figure 1).

Recently, a number of novel hydrogels such as DNA-enabled [56] and hyaluronate hydrogels [57] have been developed for diverse biomedical applications. DNA-based hydrogels as delivery carriers for gold nanoparticles (AuNPs) and the anticancer drug doxorubicin have been disclosed [58]. The DNA hydrogel degraded after laser excitation led to distribution of encapsulated gold nanoparticles and subsequent release of drug. The cytocompatibility of the proposed drug delivery system confirmed the effectiveness of a combination strategy between photothermal and chemotherapeutic approaches in cancer treatment.

**Figure 2:**
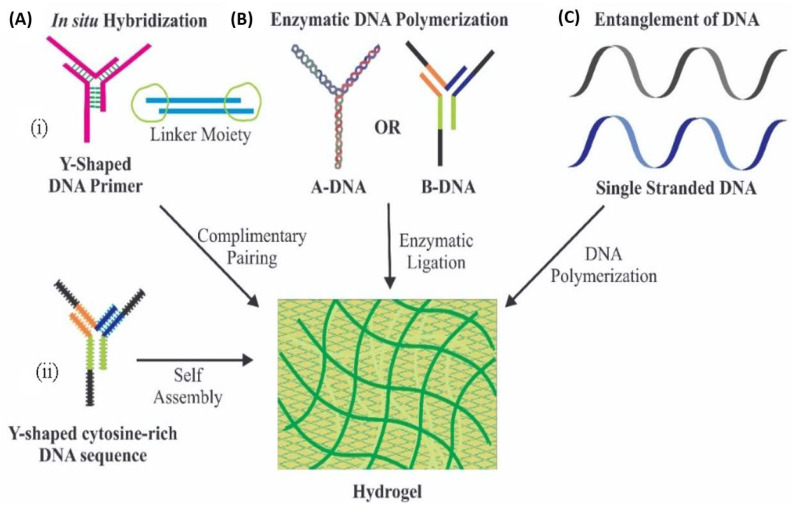
Schematic representation of the DNA hydrogels formation by various techniques. (**A**) Hybridization of DNA with its complementary strands using linker moieties (**i**) and i-motifs (**ii**); (**B**) enzymatic ligation and (**C**) entanglement of DNA.

The concept of the development of hydrogels depends on the potential of the branched DNAs to cross-link with complementary strands through network formation [59]. Several approaches have been proposed for the synthesis of hydrogels, particularly using DNA sequence (Figure 2), e.g., hybridization of DNA with its complementary strands using linker moieties, wherein DNA primers are allowed to interact with its complementary strand in order to produce a 3D network of DNA. Similarly, i-motifs, a tetrameric structure of cytosine-rich DNA sequences, which possess the potential of self-assembly, can form a four-stranded DNA complex. A second strategy is the enzymatic ligation; this technique uses enzymatic polymerase amplification reactions to join two strands of DNA. Another strategy includes entanglement of DNA; this technique utilizes application of combined rolling circle amplification and multi-primed chain amplification to synthesize long DNA strands through entanglement [60].

## 4. Role of Hydrogels in Drug Delivery Systems, Tissue Engineering, and Wound Healing

The drug delivery application of porous hydrogels can be improved and tuned by varying the cross-linking density of the gel matrix utilizing different physical and chemical cross-linking techniques. Its porosity permits entrapment of actives and the resultant release rate depends on the diffusion coefficient through the three-dimensional polymer network of the gel matrix. Surface-specific modification/grafting on polymer structures can modulate the drug flux by changing the permeability in response to external stimuli and therefore release kinetics. The biocompatibility of the hydrogel is mainly due to high water content and the physicochemical/mechanical characteristics similar to the native extracellular matrix are ideal for wound dressing. In addition, hydrogels are reasonably deformable and adaptive to the type of surface to which they are applied. The latter properties in combination with the bio/mucoadhesive nature of hydrogels can be utilized to confine them at the site of application for various biomedical applications [61]. The main disadvantages of hydrogels are their low mechanical strength in the swollen state, non-uniformity of hydrophobic drugs, rapid drug release, poor bacterial barrier, and semipermeability to gases and water vapor.

Various gelators at different concentrations or time durations can be used to impart mechanical strength to the injectable hydrogels, and/or to optimize the release rate by controlling interactions between the hydrogel and entrapped agents. The tensile strength needed from a drug-loaded hydrogel is usually based on the particular site and utilization of the hydrogel in high-stress locations such as cartilage tissue. Incorporation of hydrophobic binding sites within polymer networks with simple methods such as solid molecular dispersion would allow more loading of poorly soluble drugs while preventing drug recrystallization, when exposed to aqueous environments [62]. Vesicular carriers such as liposomes, microspheres, nanoparticles, and niosomes incorporated within the hydrogel matrix have the ability to extend and control the release of drug while eliminating or minimizing the burst release typically associated with these particle-based drug delivery systems [63]. Self-nanoemulsifying drug delivery systems constituted with bupivaccine dispersed in Carbopol gel for topical treatment of cutaneous leishmaniasis have been reported. The application of topical nano-enabled gels for a week led to drastic decrease of parasite accumulation nearly 100% comparable to the that of an intralesionally administered commercial product in a Leishmaniasis amazonensis BALB/c model [64].

There is an urgent need for the delivery of biological products such as vaccines, proteins, and hormones, which can be destabilized or change structure by interactions with hydrogel medium. Pre-entrapment or complexation with biotherapeutics before in situ hydrogel formation may address this problem to certain extent.

## 5. Drug Delivery Systems

### 5.1. Self-Assembled Nanoparticle System

Nanogels, or hydrogel nanoparticles, have gained tremendous interest as one of the most appealing nanoparticulate drug delivery systems in recent years since they combine unique hydrogel characteristics with submicron particle size. The drug molecule is either conjugated to the surface of the nanoparticles or encapsulated and protected inside the core. The surface charge, hydrophobicity, and particle size can be appropriately adjusted to avoid clearance to allow for both active and passive targeting. Further, controlled or sustained release, ability to reach small capillary vessels, penetration to tissue via para-cellular or transcellular pathway, and feasibility for administration through different routes are the other advantages of self-assembled nanoparticle systems. The basics of self-assembled nanoparticle systems are described in a recent review [65].

The polymeric nanoparticles can be efficiently utilized for the intracellular delivery of various therapeutic agents such as oligonucleotides, small interfering RNA (siRNA), DNA, and proteins. Nanoparticles are expected to uptake in cells through endocytic pathways through either specific or non-specific interactions with cell membranes. An effective delivery of a drug requires the nanoparticulate system to circumvent intracellular physiological barriers such as endosomes and deliver the drug directly within cytosol. Hydrogel nanoparticles of specific dimensions and constitutions prepared by particle replication in non-wetting templates have been explored as delivery vectors for transfection with SiRNA [66]. Amphiphilic block copolymers and peptide oligomers with distinct order can undergo self-assembly to form nanostructured hydrogels at physiological pH. Introduction of cross-linkable junctions such as hydrophobic groups, pH-sensitive moieties, and stereocomplex crystallization domains in the polymer structure can create a variety of sol-gel transition hydrogels. Functionalized hydrogels prepared through biological molecules such as heparin, cell adhesive peptides, and hyaluronic acid can furnish sustained release of therapeutic proteins or facilitate the growth and function of cells. Due to small particle size, hydrogel nanoparticles (polymeric nanogel or macromolecular micelle) have gained considerable attention as impressive drug delivery carriers.

Various methods such as ionotropic gelation, reversed phase microemulsion, emulsification solvent evaporation, nanoprecipitation, layer-by-layer coating, and self-assembly have been utilized to prepare chitosan nanoparticles [67]. Spontaneous formation of polyelectrolyte or a self-assembled polyelectrolyte complex occur when negatively charged plasmid DNA solution is mixed with cationic charged chitosan dissolved in acetic acid solution. Chitosan nanoparticles prepared by ionic gelation method using tripolyphosphate anions have shown excellent entrapment efficiency of glycyrrhizin [68]. Surface modification with PEG was found to decrease the positive charge of the particles and encapsulation efficiency. The release profile demonstrated an initial burst effect followed by sustained release due to diffusion and polymer matrix degradation. Insulin-loaded mucoadhesive alginate/chitosan nanoparticles demonstrated an encapsulation efficiency over 70% and pH-dependent release under simulated gastrointestinal conditions. Following oral administration in diabetic rats, a hypoglycemic effect was observed for over 18 h [69].

The primary reason for interest in preparing PEG-functionalized nanoparticles is to improve the long-term systemic circulation. PEGylated gelatin nanoparticles have been evaluated as an intracellular delivery vehicle for tetramethylrhodamine-labeled dextran. Results indicated that a large fraction of the PEGylated nanoparticles concentrated in the perinuclear region of the BT-20 tumor cells due to endocytosis. The presence of PEG chains decreased the percent release tetramethylrhodamine-labeled dextran in the presence of proteolytic enzyme due to steric repulsion [70].

Gelatin has shown its ability to form nanocomplexes with different polymers via ionic complexation, graft polymerization, or Maillard reaction. Gelatin-polyacrylic acid core-shell nanoparticles prepared via polymerization of anionic acrylic acid monomers showed significantly superior anticancer efficacy in hepatic H22 tumor-bearing mice in comparison with free drug [71]. Gelatin-coated lipid nanoparticles have been evaluated for bioavailability improvement of amphotericin [72], while magnetic gelatin nanoparticles were investigated for possible targeting of chemotherapeutic agents [73]. Utilization of gelatin obtained through recombinant DNA technology and use of two step desolvation techniques can overcome some of the limitations associated with gelatin such as immunogenicity and heterogenicity.

Hydrogel-based nanocarriers are used for sustained release of low-molecular-weight compounds such as adriamycin, camptothecin, cisplatin, curcumin, docetaxel, doxorubicin, paclitaxel, prednisolone, and saquinavir [74]. A combination of DOX/IL-2/IFN-g included in a temperature-sensitive polypeptide hydrogel has been developed for the effective treatment of melanoma [75]. The preparation of PELG7-PEG45-PELG7 copolymer was performed through ring-opening polymerization of g-ethyl-l-glutamate-*N*-carboxyanhydride initiated by diamino PEG. The cytocompatibility and cytotoxicity of the synthesized copolymer were determined by methyl thiazolyl tetrazolium against B16F10 cells. A flow cytometry technique was employed to analyze the apoptosis of drug-laden hydrogels against B16F10 cells. The cell cycle arrest of B16F10 cells on exposure to hydrogels loaded with drug was measured by Fluorescence-Activated Cell Sorter. The in vivo biodegradability of PELG7-PEG45-PELG7 was tested in Sprague Dawley rats. The in vivo antitumor performance of drug-loaded hydrogel was evaluated in a BALB/C mice model grafted with B16F10 cell line. The in vitro tumor inhibition against B16F10 cells with DOX/IL-2/IFN-g entrapped hydrogel demonstrated increased antitumor efficacy proved via enhanced ratio of cell apoptosis and G2/S phage cycle arrest. It was disclosed that a combined approach improved therapeutic efficacy against B16F10 melanoma xenograft probably due to increased proliferation and tumor cell apoptosis CD3þ/CD4þ T-lymphocytes and CD3þ/CD8þ T-lymphocytes. Briefly, the technique of site-specific delivery of DOX/IL-2/IFN-g utilizing the polypeptide hydrogel presented a viable technique for effective melanoma treatment.

Due to the ability to permeate the lymphatic draining system, nanoparticulate systems are apt for directing the delivery of antigens to dendritic cells and hence stimulating the T cell immunity. A glycol chitosan nanogel was functionalized with folate to enhance its intracellular drug delivery or incorporated for the asialoglycoprotein receptor used for liver targeting [76] or conjugation of monoclonal antibodies for specific cell surface markers [77].

**Figure 3:**
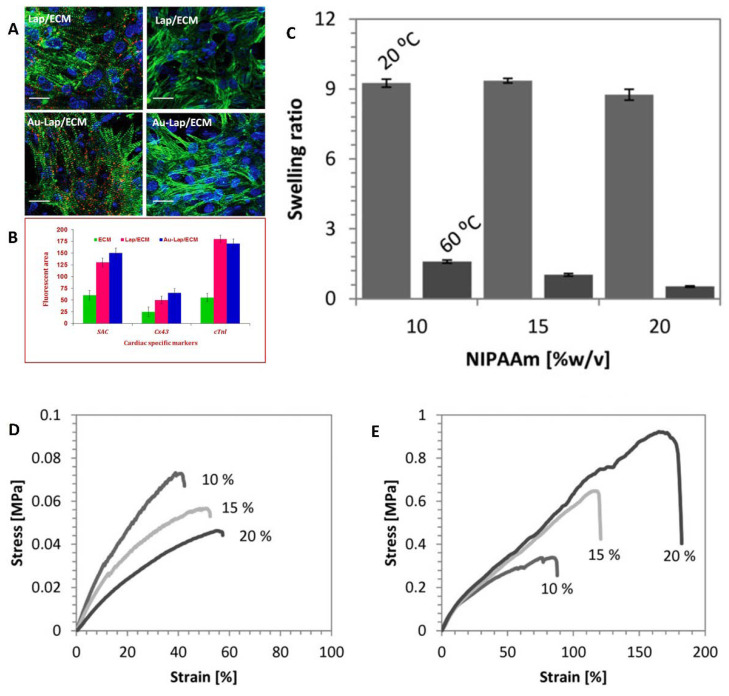
(**A**) Cytoskeleton arrangement of cardiomyocytes under cardiac-specific markers. (**B**) Bar graph of covered area representing immunofluorescence images of cardiomyocytes [78]. (**C**) Swelling ratios of alginate/PNIPAAm ICE hydrogels with varying concentrations (10%, 15%, and 20% (*w*/*v*)) of N-isopropylacrylamide concentrations at 20 and 60 °C. (**D**) Tensile stressstrain curves of alginate/PNIPAAm ICE hydrogels at 20 °C. (**E**) Tensile stress–strain curves of alginate/PNIPAAm ICE hydrogels at 60 °C [79].

Incorporating biocompatible nanoparticles tino the hydrogel matrix can significantly increase cell adhesion and therapeutic potential of the hydrogels. An injectable hydrogel containing gold and laponite nanoparticles was tested to improve the clinical efficacy of cardiovascular regeneration [78]. It was suggested that incorporation of electroconductive materials with nanoparticles in the myocardial extracellular matrix (ECM) may increase the remaining functional characteristics of cardiomyocytes. The modification of laponite clay was carried out using cetyl trimethylammonium bromide and ionic liquids by ion exchange method. The hydrogels were fabricated by mixing laponite nanosuspension with ECM solution for one hour at 37 °C. The gold nanoparticles were prepared by adding tetrachloroauric acid and tri-sodium citrate solution to the above mixture under stirring for 15 min. It was demonstrated that the cell survival percentage of the treated cells with Gold-Lap/ECM hydrogel (Figure 3) was more than Lap/ECM matrix, which confirmed that inclusion of gold nanoparticles provided adequate aid for cell activity.

### 5.2. Buccal Delivery

A buccal drug delivery system has attracted keen attention since the buccal mucosal membrane is more permeable and allows rapid permeation of pharmaceutical actives into the systemic circulation. An ideal polymer employed for buccal delivery should have excellent spreadability, swelling, rheological characteristics, adequate bioadhesivity in dry and wet state, mechanical strength, and be non-toxic, economical, biocompatible, and biodegradable [80]. To limit the release and permeation of the drug across the buccal mucosa, hydrogel-based tablets, films, or bioadhesive patches can be successfully developed. Hydrogel present in mucoadhesive tablets can modulate the release pattern of the drug depending on the hydration rate and subsequent swelling process of the construct. The utilization of cellulosic or acrylic polymers typically results in prompt and prolonged bioadhesion even with high drug entrapment. Frequently used hydrogel-based polymers for buccal delivery dosage forms are hydroxyethyl cellulose (HEC), carboxymethyl cellulose (CMC), hydroxypropyl methylcellulose (HPMC), hydroxypropyl cellulose (HPC), chitosan, polyacrylic resins and polyvinyl alcohol (PVA), polyvinyl pyrrolidone, Kollicoat, maltodextrin, Lycoat NG 73, and pullulan [81,82].

Buccal films prepared from mucoadhesive polymers like chitosan-carbopol/chitosan-gelation loaded with miconazole nitrate demonstrated excellent in vitro efficacy against Candida species. Further, buccal films provide more flexibility, increase contact time, and offer more protection to the wounded oral mucosa. Bioadhesive patches were fabricated from various ionic (sodium CMC and chitosan) and non-ionic (PVA, HEC, HPMC) mucoadhesive hydrogel polymers [83,84,85]. Nanocarriers loaded into hydrogels are used for buccal delivery to improve the residence time and bioavailability, and to protect the drug from degradation [86].

**Figure 4:**
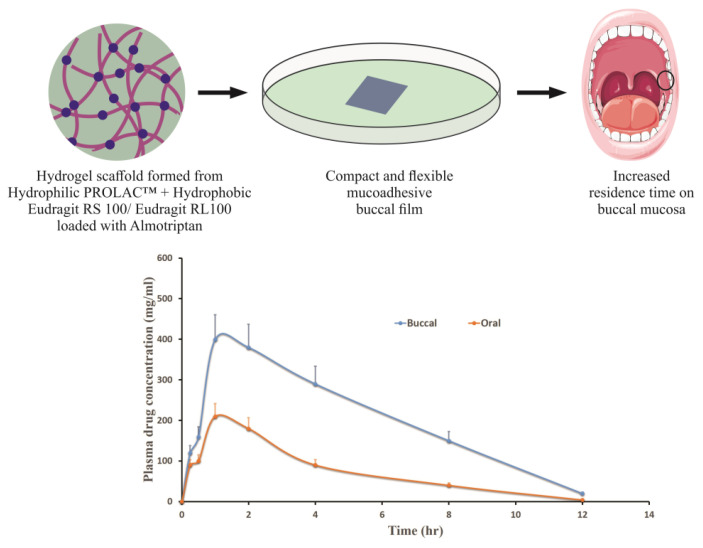
Enhanced buccal permeation demonstrated by the hydrogel-based mucoadhesive almotriptan buccal film compared to oral suspension containing equivalent dose [80].

Evaluation of mucoadhesion using tensile strength and rotating cylinder tests revealed CMC, HEC, and HPMC as the most promising cellulose derivatives for buccal application. Thus, generally regarded as safe approved cellulose-based patches can be utilized for different conditions affecting the intraoral cavity [87]. The PROLOC™ bioadhesive drug delivery system attaches remarkably well to the soft, hydrated mucus membranes of the body since it is formed from starch-polyacrylic acid blends, which then entirely erode and disappear. We have effectively utilized Proloc 15 polymer along with water-insoluble polymers, Eudragit RL100/RS100, in order to develop a buccal film for the effective delivery of almotriptan [80]. Enhanced buccal permeation was demonstrated by the developed hydrogel-based mucoadhesive film compared to oral suspension with equivalent dose (Figure 4). A dental light-curable gelatin hydrogel containing antimicrobial peptide showed significant bioadhesivity to physiological soft tissue such as gingiva as well as hard surfaces such as bone or titanium implants. Further, cytocompatibility, biodegradability, and tissue regenerative capability demonstrated by these multifunctional hydrogels demonstrate its potential ability to be utilized in various peri-implant diseases [88].

When pH of the exposed medium is more than the pK_a_ of the negatively charged polymer chain, hydrogels are dissociated and subsequently swollen. Cationic polymers possessing amino acid functional groups show significant aqueous solubility at acidic pH and poor aqueous solubility at neutral pH. Better protection of labile drug was observed in pH of oral cavity utilizing cationic hydrogel polymers [89].

Development of a buccal drug delivery system employing a catechol-functionalized chitosan hydrogel cross-linked with non-toxic biocompatible cross-linker genipin has been described. The gelation time and other mechanical properties of this hydrogel were comparable to chitosan. In vivo studies indicated sustained release of lidocaine up to 3 h without any untoward effects on rabbit mucosa [90]. The main disadvantage of genipin cross-linked film is the formation of a dark blue color in the cross-linked film.

Cytokine PharmaSciences Inc. has recently developed the Pilobuc buccal insert based on a hydrogel polymer technology that allows sustained release of pilocarpine for several hours. The novel proprietary buccal formulation minimizes many undesirable side effects associated with oral systemic administration in case of xerostomia and Sjögren’s Syndrome. The US Food and Drug Administration (FDA) has recently approved OraDisc A, a proprietary CMC- and HPMC-based mucoadhesive patch of amlexanox used for the treatment of aphthous ulcers.

### 5.3. Oral Delivery

Hydrogels employed for oral therapeutic systems should possess ideal properties such as biocompatibility, capacity to accommodate diverse actives, tunable properties, site-specific delivery, and controlled release of synthetic drugs and biotherapeutics for both local and systemic treatment applications.

In oral drug administration, the greatest challenges faced by the formulation scientist is to deliver hydrophilic macromolecules such as insulin or heparin. Hydrogels are well adapted to accommodate these drugs in their polymeric networks to protect them from the acidic conditions of the stomach. To achieve this target, natural polymers with anionic pendant groups are grafted with acrylic acid derivatives to obtain stimuli-sensitive behavior in the final hydrogel polymer. Various polymeric network design strategies to create smart hydrogels with prompt responsiveness and improved mechanical properties for diverse applications have been reviewed elsewhere [91].

Various investigations have revealed the effectiveness of the pH-sensitive hydrogels included in oral dosage forms of chemotherapeutic agents, insulin, calcitonin, and interferon-β [89,92]. Particle size of the hydrogel can be tailored to deliver the drugs to either targeted sites (e.g., 1–1000 µm-small intestine), intracellular vesicles (e.g., 50–200 µm-endosomes), or lymphatic vessels (<30 µm). It was reported that orally administered chemotherapeutics are more effective, and have minimum side effects when compared to parenteral administration [93].

Superporous hydrogel (SAH) formulations possess good mechanical strength and faster swelling rates, suggesting them appropriate for oral drug delivery and biomedical applications [94]. To achieve the goal of acid protection and intestinal release, anionic hydrogels are typically used since they are ionized, swollen, and have increased pore size and hence allow drug release at a pH higher than the pKa of the polymer chain [95]. Ionization at a pH lower than the pKa of the polymer chain would allow cationic hydrogels for drug delivery in stomach and intracellular environments. A new class of amphiphilic polymeric carriers composed of anionic P(MAA-g-EG) incorporated with PMMA nanoparticles for oral delivery of low- molecular-weight proteins or hydrophobic drugs such as doxorubicin for targeted delivery to the colon have been reported [96]. It was demonstrated that increased embedding of nanoparticles leads to high drug entrapment and extended release to the colon. Similarly, spray dried gelatin nanospheres showed greater mucoadhesion following oral administration [97].

A novel hydrogel fabricated from grafting polycaprolactone onto a methacrylic acid copolymer structure was utilized for the oral delivery of amifostine. The radio-protective efficacy evaluated through complete blood parameters and a 30-day survival study in mice indicated effective radioprotection. Such hydrogels could protect the drug from acidic and enzymatic degradation in the stomach to deliver the drug to the intestine [98]. From the results of high encapsulation efficiency (94.2 ± 2.6%), drug-loading capacity (13.5 ± 0.4%), and extended in vivo hypoglycemic effect (~24 h), the authors concluded that insulin entrapped in lectin-functionalized carboxymethylated *kappa*-carrageenan microparticles has the capacity to develop as an oral insulin drug delivery system [99].

Forming polyelectrolyte complexes with natural anionic alginate polymer matrices and chitosan can control the drug release profile as well as inhibit the rapid decomposition of alginate at alkaline pH. Interpenetrating networks of such complexes have shown promise for proteins and vaccine delivery. The potential ability of novel biocompatible hyaluronic acid derivatives designed for protein and peptide delivery was evaluated using α-chymotrypsin under simulated acidic conditions [100].

Intracellular chemotherapeutic delivery of hydrogel carriers has been carried out with acid labile oxime linkage or acetals. The temperature- and pH-sensitive, acid degradable carbohydrate-based nanogels for the endosomal delivery of DNA and enzyme have been investigated [101]. Novel pH- and temperature-responsive hydrogels synthesized from *N*-Acryloyl-l-phenylalanine amino acid and guar gum polymer via free radical polymerization has been suggested for controlled release of an antineoplastic agent, imatinib mesylate [102]. The advantages of hydrogels are stimuli responsiveness, biocompatibility, and high drug loading while the main limitation is complete dependence of swellability on diffusivity of water. Hydrogel polymers suggested for oral protein and peptide delivery are PMMA, alginate-based, and chitosan-based hydrogels [103].

**Figure 5:**
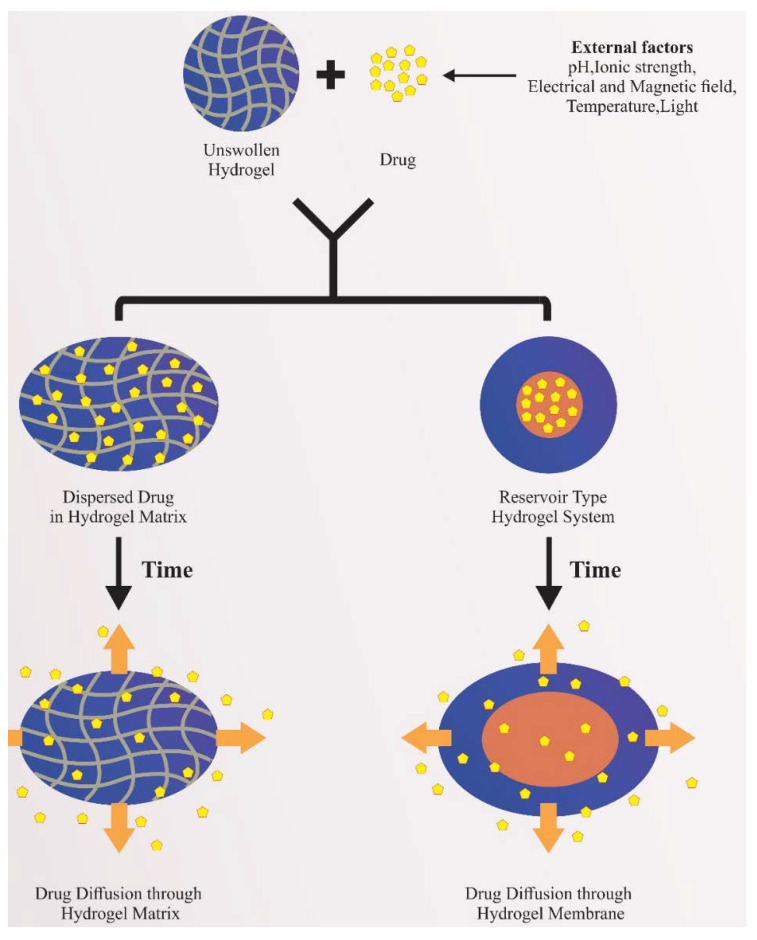
Conventional hydrogel oral preparation methods and drug release behavior.

Conventional hydrogel oral preparations are generally prepared as matrix or reservoir systems (Figure 5). In a matrix system, the dispersed drug diffuses out of the matrix after the exposure to aqueous medium and subsequent dissolution of the matrix. In the hydrogel reservoir systems, the drug delivery rate is mainly influenced by the physicochemical characteristics of the drug and polymer, as well as the thickness of the polymer shell. The mechanisms of drug release from hydrogels are mainly influenced by dosage form-related variables and/or polymer characteristics. On the other hand, nanosuspension incorporated into the hydrogels for oral therapy also shows significant improvement in bioavailability [104]. Selected examples of commercial dosage forms for oral delivery are summarized in Table 3.

### 5.4. Transdermal Drug Delivery

Transdermal drug delivery offers an important means of delivering drugs through the surface of the skin for local or systemic effects. The advancement of transdermal drug delivery has been sharply limited by the failure of many drugs to permeate the skin at clinically relevant rates, for instance proteins and peptides. Both chemical and physical approaches are used to enhance the transdermal permeation of actives [105]. Hydrogels suitable for transdermal drug delivery systems should possess excellent biocompatibility, biodegradability, elasticity, non-allergenicity, ease of application, soft consistency, and high water content. Due to the hydration effects induced on the skin, hydrogels are utilized to increase the transport of drugs across the skin [106,107].

Microneedle arrays fabricated from cross-linked hydrogel polymers upon insertion into the skin can absorb interstitial fluid to form continuous channels connecting to dermal circulation. In the first reported study, an aqueous mixture constituting poly (methylvinylether/maleic acid) and PEG 10,000 was used to develop microneedles employing silicone micromold templates. It was demonstrated that hydrogel-based microneedles provided prolonged transdermal drug administration and the release rate of the drug is mainly influenced by the cross-link density of the hydrogel polymer. Furthermore, pulsatile or bolus delivery can be electrically modulated and the method requires only minimally invasive patient monitoring [108]. The feasibility of hydrogels forming a microneedle patch for the sustained delivery of high dose metformin has been investigated [109]. After the hydrogel microneedle application, steady state plasma drug concentration (3.2 μg/mL) was maintained until 24 h in rat model with an overall bioavailability improvement of nearly 30%. Thus, hydrogel-forming microneedle technology could be successfully adopted for sustained release delivery of therapeutics in near future. It was reported that iontophoretic delivery of methotrexate from hydrogel was found to be more effective compared to passive delivery, signifying the potential of hydrogel-based iontophoresis [110]. The repulsion between cationic buprenorphine and chitosan vehicles may account for the difference in permeation rates from various vehicles [111]. A novel portable and disposable iontophoretic reverse electrodialysis device (RED) connected to a electroconductive hydrogel constituted of polypyrrole-embedded PVA has been developed [112]. It was concluded that charge-inducing agents through RED-driven iontophoretic units can effectively facilitate the transdermal transport of both acidic and basic drugs.

Due to high water content, swollen hydrogels can provide ease of application and more comfort for patients compared to transdermal patches. In our laboratory, an optimized gel constituting nebivolol with gellan gum, carbopol, and PEG 400 was developed for transdermal delivery and subsequently evaluated in albino rats. Considerable enhancement of transdermal flux (30.86 ± 4.08 μg/cm^2^/h) was noticed in optimized gels compared to nebivolol suspension [113]. Therefore, stable hydrogels formed from the combination of gellan gum and carbopol 940 could be successfully utilized for transdermal delivery of biopharmaceutical classification system class II drugs.

A paintable oligopeptide hydrogel containing paclitaxel-encapsulated cell penetrating peptide-modified transfersomes was developed for topical melanoma treatment. A plastered patch comprising a paclitaxel-modified transfersomes hydrogel above the melanoma tumor provided extended retention capacity and significant suppression of tumor growth in combination with systemic chemotherapy using taxol [114]. Various studies have evaluated the role of hydrogels in promoting the dermal drug delivery via nanoparticulate carriers [115].

The potential capabilities of three nanocarriers such as polymeric micelles, solid lipid nanoparticles, and self-nanoemulsifying drug delivery system (SNEDDS) were compared as transcutaneous drug delivery systems for lidocaine, through an artificial skin membrane [116]. Highest lidocaine loading was noticed in SNEDDs compared with polymeric micelles and solid lipid nanoparticles while cumulative lidocaine concentration after 6 h for both polymeric micelles and SNEDDS was significant compared to solid lipid nanoparticles.

In another study, significant dermal delivery of hydrocortisone from novel composites constituted of micelles and hydrogel was reported. An enhanced permeation rate with cumulative quantities of hydrocortisone transported was many folds higher compared to hydrocortisone cream [117]. Thus, incorporating a particulate carrier loaded with the drug within the hydrogel matrix can be considered as a feasible and effective strategy for transdermal therapy.

The pH-sensitive potential of Eudragit S100 nanoparticles loaded with piroxicam was evaluated for transdermal delivery using Carbopol 934. Nanoparticles (25–40 nm) were prepared by a simple nanoprecipitation technique and dispersed in a Carbopol 934 hydrogel. Results indicated that delivery of these nanocarriers from the hydrogel matrix exhibited considerable enhancement of piroxicam via mice skin [118]. Transdermal enhancement potential of hydrogel led to commercialization of products such as Voltaren Gel^®^, Lidoderm^®^ patch, and Persa-Gel^®^.

### 5.5. Vaginal Drug Delivery

The vaginal route is mainly used for the treatment of conditions and diseases affecting the vulvovaginal area caused by yeast, mold, fungi, and bacteria. The vaginal mucosal lining is also convenient for the absorption of diverse drugs including proteins and peptides for systemic effects, since it offers a high surface area and contains an extensive network of blood vessels. The chemical structure and properties of the most frequently used polymers for vaginal drug delivery applications and recent advances in hydrogels as drug delivery vehicles for vaginal infections have been reported [119].

Mucoadhesive chitosan and sodium alginate were successfully utilized to fabricate vaginal inserts loaded with chlorhexidine digluconate [120]. The inclusion of these polymers in the formulation was to impart better adhesion between the device and the mucosal lining of the vaginal cavity.

The interaction of the polymer with mucin is related to the physicochemical properties such as molecular weight, porosity, functional groups, molecular conformation, swelling and cross-linking capacity, and degree of cross-linking. A novel readily spreadable bigel was developed with a combination of hydrophilic carbopol 934 gel with organogel constituted of sorbitan monostearate-sesame oil. In vitro studies indicated enhanced metronidazole release rate and antimicrobial efficacy compared to marketed Metrogyl^®^ gel [121].

The design of hydrogel-based dosage forms depends on the route of administration; for instance, in vaginal administration the dosage form is usually either cylindrical or torpedo fabricated devices. A vaginal drug delivery system was prepared with flexible propylene glycol-constituting liposomes loaded with metronidazole or clotrimazole dispersed in carbopol hydrogel. It was found that penetration of deformable propylene glycol liposomes in hydrogel was more rapid than liposomal preparations without hydrogel. In vitro studies from the drug-loaded liposomes in hydrogel under simulated vaginal conditions indicated sustained and diffusion-controlled drug release [122].

Vaginal hydrogels containing antimicrobial agents could act as a physical and therapeutical barrier against viral infections and restrict the spreading of the virus through the vaginal mucosa. For instance, Pluronic^®^ F127 and HPMC containing carboxy group modified polystyrene particles was found to decrease the motility of Type 1 human immunodeficiency virus (HIV-1) by changing the size and surface charge [123]. Furthermore, the loading of anti-HIV CD4 M48U1 mini-peptide within these polymers did not modify its anti-HIV-1 activity, unlike conventional hydrogel.

An improved mucoadhesion was observed in a hydrogel combination made of HPMC (5% *w/w*) and chitosan loaded with metronidazole nanoparticles. The viscoelastic behavior of the developed product was similar to commercial Zidoval^®^ vaginal gel [124]. An interesting investigation on the hydrogel composed of low-molecular-weight chitosan revealed a significant reduction of the metabolic activity during the development of the biofilm and decreased total biomass to ~85% [125]. The drastic reduction of structural complexities of biofilm contributed to by low-molecular-weight chitosan can be effectively utilized for the treatment of vulvovaginitis. Nanocapsules loaded with indole-3-carbinol thickened with gellan gum demonstrated pseudoplastic flow characteristics, vaginal mucosal adhesion, neutral pH, non-irritating behavior, and enhanced in vitro anti trichomonas vaginalis activity [126].

A novel delivery system prepared from post-expansile hydrogel foam aerosol of propylene glycol-liposomes constituting matrices for vaginal drug delivery applications has been reported. Results from this investigation revealed uniform distribution over the vaginal epithelium, better adhesion properties, and increased contact time with the vaginal mucosal membrane [127]. In order to overcome the limitations of low solubility and poor bioavailability of resveratrol or epicatechin, a vaginal system comprising liposomal polyphenols in chitosan-based hydrogel was developed. The in vitro release study demonstrated sustained release and anti-inflammatory and excellent free radical scavenging activity in comparison to antioxidants vitamin C and E [128].

### 5.6. Ocular Delivery

The ophthalmic drug delivery system is challenging for formulation scientists due to unique anatomical, physiological, and biochemical barriers of the eye. Hydrogel polymers selected for ocular delivery should be biocompatible, biodegradable, and non-cytotoxic with prolonged retention time. Hydrogels as drug delivery carriers offer several advantages in ophthalmic applications such as ease of preparation, non-invasive administration method, and high water content, which may stabilize the potency of bioorganic actives such as peptides, nucleic acids, and proteins [129]. Besides, hydrogels can entrap large doses of drugs in their three-dimensional porous molecular network also given through via small gauge syringes. Hydrogel polymers and cross-linkers used in ocular formulations and their associated chemistries are extensively reviewed elsewhere [130].

**Figure 6:**
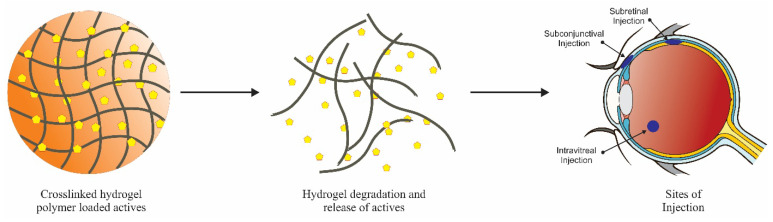
Different targets and potential ocular delivery routes available for drug-loaded hydrogel polymers.

Different delivery routes and target sites for cross-linked polymers are presented in Figure 6. Due to short elimination half-life, Avastin^®^ used in age-related macular degeneration is administered as repeated injections. The frequency of dosing and loss of drug by drainage can be overcome by formulating in hydrogel. An injectable polysaccharide cross-linked hydrogel synthesized by mixing glycol chitosan and oxidized alginate aqueous solution has been reported [131]. An in vitro release study indicated rapid Avastin^®^ release within 4 h followed by sustained release up to 3 days. The authors suggest that the developed in situ gel with controllable degradation rate might be utilized as a versatile carrier in ocular delivery.

Thermoresponsive hydrogels are the most investigated hydrogel polymers because the temperature differences existing outside and inside the body are sufficient enough to cause cross-linking of the polymer or promote the drug release. The preparation of a sustained release, latanoprost-loaded injectable thermosensitive hydrogel system constituting chitosan/gelatin/glycerol phosphate demonstrated rapid gelation within 1 min at 37 °C. In vivo evaluation in a rabbit glaucoma model showed significant reduction of intraocular pressure after a single subconjunctival administration of chitosan/gelatin/glycerol phosphate [132]. Another thermosensitive in situ gel prepared from triblock polymer poly-(dl-lactic acid-*co*-glycolic acid) (PLGA)–PEG–PLGA was tested for ophthalmic delivery of dexamethasone acetate. It was synthesized through ring-opening polymerization using DL-lactide and glycolide in the presence of PEG (PEG1000:PEG1500 = 1:1) and the catalyst tin (II) octoate. The copolymer incorporated with 20% *w/w* of dexamethasone had a low critical solution temperature of 32 °C, which is close to the ocular surface temperature. Significant improvement of ocular bioavailability was indicated by higher C_max_ (125 ng/mL) and area under the curve (AUC; 436.0 ± 52.60 ng·h/mL) in rabbits [133]. The PEG diacrylate cross-linked hydrogels developed from PNIPAAm appears to be a promising, minimally invasive platform for extended drug delivery to the posterior segment [134]. The prepared hydrogel retained better thermoresponsive characteristics since it can absorb more water below lower critical solution temperature through homogeneous macropores created by PEG. In another study with the same polymer, it was found that the rate of protein (bovine serum albumin, immunoglobulin G, bevacizumab and ranibizumab) release can be controlled by varying the cross-linker density without opaqueness, which may otherwise interfere with visual function [135]. Short duration of action and incomplete biodegradability are the few issues concerning this novel drug delivery platform.

The key advantages of iontophoresis are the significant enhancement of drug release and control of therapeutic compounds including large-molecular-weight drugs. A pulsed low current (1.6 mA/cm^2^) non-invasive iontophoretic treatment using methotrexate-loaded hydrogels was reported to be potentially effective in treating ocular inflammatory diseases and intraocular lymphoma [136]. After iontophoretic treatment, s minimum effective drug concentration was maintained for at least 8 h at the sclera and retina and for 2 h at the aqueous humor.

The sustained release preparations can facilitate even small-molecular-weight actives that may usually eliminate fast from the vitreous humor, particularly from intravitreal solution dosage form. A thermoresponsive PLGA-PEG-PLGA hydrogel was demonstrated to prolong the delivery of Avastin^®^ up to 14 days in the vitreous humor and retina following intravitreal injection. The drug load in the polymeric hydrogel did not transform the sol-gel behavior and exhibited no apparent toxicity to retinal tissues [137]. It was found that after the intravitreal injection, the aqueous humor concentrations of Avastin^®^ from aqueous solution of Avastin^®^ and the Avastin^®^ hydrogel at 1 day after drug administration were 674.69 ± 297.84 ng/mL and 4471.10 ± 386.34 ng/mL, respectively. It was suggested that the extended retention (8 weeks) in the corneal tissue is probably due to corneal composition of collagen without enzymes, resulting in slow clearance.

Intracameral delivery of drugs permits drug diffusion into anterior chamber and a few posterior ocular tissues. A biodegradable in situ gelling drug delivery system containing pilocarpine prepared from carboxylic acid end capped-PNIPAAm for intracameral administration of glaucoma has been reported. Ocular bioavailability and duration of action was found to be more compared to ophthalmic drops, pilocarpine injection, or drug-loaded PNIPAAm [138]. Some commercial examples of hydrogel-based ocular drug delivery systems and soft contact lens (SCL) materials are summarized in Table 4.

Biodegradable PLGA microspheres containing the anti-vascular endothelial growth factor aflibercept for ocular drug delivery have been disclosed [139]. The microspheres were dispersed within a thermosensitive hydrogel made up of biodegradable PEG-*co*-(l-lactic acid) diacrylate/NIPAAm hydrogel. In vitro release demonstrated an extended and controlled drug release (0.07–0.15 µg/day) for up to 6 months.

Due to beneficial physicomechanical properties such as wound leakage (>80 mmHg), duration of cross-linking (<30 s), bioadhesive strength (0.1 kPa), mechanical strength (5–200 kPa), percentage swelling (<200% *w*/*w*), decomposition, and retention time (1 week to 6 months), hydrogels represent an important substitute for wound closing techniques such as sutures or stromal hydration [140]. An example of an FDA-approved ocular healing hydrogel for corneal application is ReSure^®^ sealant prepared from four armed *N*-hydroxysuccinimide-capped PEG cross-linked trilysine amine. Duraseal^®^ was approved for cranial adhesion and investigated for wound sealing [141]. These biocompatible formulations, in addition to sealing corneal wounds, restore and maintain intraocular pressure similar to normal healing process, avoid astigmatism, and are removed during re-epithelization.

Various hydrogel polymers are being evaluated to find an alternative for vitreous substitutes including HPMC, hyaluronic acid/gellan gum, pHEMA, poly (glycerol methacrylate), siloxanes, PVA, and PVP [129]. The clinical success of vitreous substitutes are limited by various issues including immune response initiation, faster assimilation or decomposition, or failure to form a complete retinal tamponade [142]. The challenging problems such as biocompatibility, sophisticated drug encapsulation and release mechanisms, process scale-up, shelf-life, and sterilization must be solved before the therapeutic applications of hydrogels can be fulfilled.

### 5.7. Contact Lens

Contact lenses provide a safe and effective way to correct vision, when used with personal care and proper supervision. Insertion of traditional SCL pre-soaked in drug solutions or post application of eye drops have the ability to release drugs, decreasing elimination and sorption across conjunctiva. Various types of polymeric hydrogels frequently employed in contact lens-based drug delivery systems, and their merits and demerits, are described elsewhere [143].

Polymeric hydrogels such as pHEMA are extensively used as SCL materials for ocular delivery of various drugs [144]. An improved biocompatibility and drug entrapment without compromising viscoelasticity was observed when it was copolymerized with 4-vinylpyridine and *N*-(3-aminopropyl) methacrylamide [145]. An artificial intraocular lens can address the issues due to cataract and myopia to restore the original visual function. Siloxane-based hydrogels and hydrophilic and hydrophobic acrylate polymers are the newer classes of intraocular lenses.

**Figure 7:**
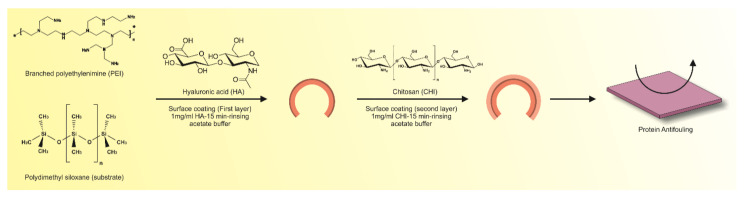
Surface-coated silicone hydrogel with hyaluronic acid/chitosan having protein antifouling characteristics as a contact lens material.

A number of researchers have employed polymerization reactions to entrap drugs, proteins, and cells in hydrogel structures. However, dissolving the drug in a polymerization mixture may result in undesired reactions causing loss of their functionality. To overcome the disadvantages, polymeric hydrogels encapsulated with colloidal drug particles have been utilized as potential ophthalmic drug delivery systems. The silica stabilized drug-loaded microemulsions were loaded in contact lenses fabricated from pHEMA hydrogel. The data revealed sustained release of the drug for eight days and the release rate could be controlled by the particle size and drug loading [146]. A ring implant was developed from timolol-loaded ethyl cellulose sandwiched between a hydrogel contact lens prepared from the same polymer. In vivo pharmacokinetic studies indicated an increase in mean residence time and AUC while a pharmacodynamic study demonstrated sustained release results in decrease of intraocular pressure for about a week [147]. Though pHEMA exhibits the resistance to crack propagation and possesses tunable mechanical properties, one of its major disadvantages is slower biodegradability that limits its application in biological fields. Researchers have been able to improve the mechanical properties and low-molecular-weight products after degradation by changing the degree of cross-linking with various polymers. Multilayer modification technique has been adopted to modify silicone hydrogel intraocular lenses with hyaluronic acid/chitosan polyelectrolyte complexes in order to prevent cell adhesion and proliferation without compromising optical characteristics [148]. A schematic overview of this preparation method is depicted in Figure 7.

A hydrogel containing a cationic functional group in its side chain was prepared with 2-hydroxyethyl methacrylate (HEMA) and methacrylamide propyl trimethylammonium chloride [149]. It was capable of entrapping an anionic drug, azulene, based on ion exchange reaction. Similarly, hydrogel contact lens materials having phosphate side groups in polymer networks were investigated for their role in cationic drug delivery. One of the most extensively studied molecular complexations is the interaction between ligands and cyclodextrin to form reversible inclusion complexes [150]. A successfully grafted acrylic hydrogel constituting β-cyclodextrin (β-CDs) exhibited enhanced capacity to entrap drugs and improved the extending of drug release in lacrimal fluid for half a month [151]. In the polymer structure, β-CDs are not a part of the chain but project on 2–3 ether bonds through the hydroxyl groups. The pendant did not change the physicomechanical properties of the polymers such as light transmittance, glass transition temperature, swelling degree, viscoelasticity, oxygen permeability, or surface contact angle. However, a cytocompatible acrylic hydrogel increased the drug loading (1300%) and drug affinity (15-fold), and decreased the friction coefficient (50%).

Molecularly imprinted polymer hydrogels are synthetic polymers displaying specific molecular recognition domains generated during polymerization reactions due to the inclusion of molecular templates of interest. The drug release from molecularly imprinted hydrogels made of methyl methacrylate functional polymer with ethylene glycol dimethacrylate was studied at different pH [152]. High drug loading capacity with desired profile following Fickian type of drug release kinetics was observed. pHEMA imprinted with norfloxacin was investigated as contact lens materials for ocular drug delivery [153]. Some examples of molecularly imprinted polymers impregnated with timolol are: *N,N*- diethylacrylamide and HEMA, natural receptor-based poly(AA-*co*-AM-*co*-HEMA-*co*-PEG200DMA), poly(AMco-HEMA-*co*-PEG200DMA), poly(AA-*co*-AM-*co*-NVPco-HEMA-*co*-PEG200DMA), and poly(AA-*co*-HEMAco-PEG200DMA). A novel antibacterial contact lens material made of an antimicrobial metal organic framework, [154] (AGMNA) incorporated into polymer hydrogel using the polymer HEMA, has been reported [154]. Significant antimicrobial activity against both Gram-negative and Gram-positive microorganisms was demonstrated.

Though the results from various investigations reveal that the drug entrapment capacity is enhanced, the affinity towards a template molecule is the main limitation of the imprinted hydrogels. Maximal drug loading is restricted by the template molecules and functional monomers of the imprinted hydrogel. In addition, the deformation of the imprinted hydrogel utilized for the contact lenses materials may affect the release profile of drugs. Molecularly imprinted materials capable of specific recognition of the template in aqueous environments with minimum nonspecific interactions with drugs and other excipients are the main focus of current investigations in this field. The synergistic combination of imprinting and external stimuli responsiveness may have tremendous practical applications in different types of drug delivery systems. Molecularly imprinted polymers are extensively probed in hydrogel contact lenses for improving drug entrapment as well as prolonging their release duration. A layer-by-layer coating of natural polysaccharides using genipin as a cross-linker to sustain the release of diclofenac sodium from the surface of silicone hydrogels and SCL has been reported [155]. A biocompatible, hydrophilic, and non-toxic coating had minor effects on the physical characteristics of SCL and successful sterilization of these biopolymers can also be carried out through a high hydrostatic pressure sterilization method. Such mono or multifunctional imprinted hydrogels as SCL materials could be potentially used in ocular drug delivery systems.

### 5.8. Wound Dressing

The complex biological process of wound healing in human beings is culminated through well-defined events, namely: coagulation and hemostasis; inflammation, chemotaxis, and activation; proliferation, maturation, and remodeling. Hydrogels as a wound dressing match most of the desirable features expected for wound healing and are the most appropriate material for burn patients. Due to the hydrogels’ unique advantages of retaining desired moisture level at the wound zone, allowing proper oxygen and moisture exchange between the wound and the surroundings, biocompatibility, possessing tissue like structure, ease of application due to softness, elasticity and flexibility, providing cooling sensation for patient’s comfort, ability to absorb serous discharges from lesions, and reduced interferences with wound healing process, their role as the preferred wound dressing is well accepted by clinicians and biomaterial scientists [156]. The poor mechanical stability of hydrogels has been resolved by using composite or hybrid membranes systems employing physical or irradiation cross-linking methods. The cross-linked polymers have the ability to retain more water in their three-dimensional mesh structures. The hydrogel structure can embed actives such as antibiotics in the molecular network during gel formation and release it in a controlled manner after application to the wounded site. Treatment of wounds in the moist environment of hydrogels has shown to enhance re-epithelialization and additionally provides a microenvironment enabling healing without scarring. Recent developments including synthetic processes of hydrogels and their biomedical applications as wound dressing materials are reviewed in the literature [157].

The material used for wound dressing is routinely subjected to: testing for tensile strength using a texture analyzer; cytotoxicity tests for biocompatibility based on human polymorphonuclear neutrophils to trigger a respiratory burst; microbial testing for antibacterial efficacy using the colon-drip flow reactor model employing the methicillin-resistant staphylococcus aureus or pseudomonas aeruginosa biofilm growth; toxicity and skin sensitivity evaluation. Permeability of moisture and gases through the film for wound dressing is essential to keep the wound comfortable and enhance the healing process. It can be evaluated using the water vapor permeability testing based on the method described in United States Pharmacopoeia.

Technological advances in moist wound dressings are evolving with different inbuilt properties such as absorption, hydration, and antibacterial activity. In order to create a moist wound environment, the application of a moisture-retentive dressing may include one or more of the following materials such as hydrogel, petroleum jelly, hydrocolloid dressing, gelling alginate dressing, film dressing, and occlusive foam dressing depending on the type of wound. Gamma radiation induced cross-linking with aqueous solutions of hydrophilic polymers such as PVA or PVP and resulted in concurrent generation of sterilized hydrogels currently marketed as “Áqua-gel” wound dressings [158]. An inexpensive method has been invented to prepare sterilized hydrogels by mixing an aqueous solution of suitable hydrophilic polymers and a cross-linking process is accomplished by thermal curing under high pressure followed by autoclaving or microwave irradiation [159].

Hydrogels have been included in the architecture of some wound dressings together with other added constituents, forming composite products beneficial for many types of wounds. Composite hydrogels made of gelatin-grafted dopamine/chitosan/carbon nanotubes [160], lignin-chitosan-PVA [161], or nanocurcumin loaded *N,O*-carboxymethyl chitosan/oxidized alginate hydrogel [162] provide new hope for efficient wound care management.

Irregularly shaped amorphous hydrogel dressings are primarily formulated to rehydrate or to create a favorable moist condition that supports healing of wounds, burns, and tissue damages caused by radiation. Selected examples of various commercial hydrogel wound dressings are displayed in Table 5. A topical anesthetic hydrogel containing lidocaine and hyaluronic acid is recommended for partial or full thickness wounds and painful dressing changes. Soft and conformable alginate dressings are particularly recommended for wounds with excessive exudates such as pressure ulcers and infected wounds. Hydrocolloid wound dressings usually composed of gelatin or pectin cellulose derivatives are recommended for partial and full thickness wounds that require a secondary dressing. Recently, an amidated pectin oxidized chitosan has been suggested as a potential wound dressing material for skin rejuvenation [163]. The main advantage of this method is its ability to form in situ gel without any cross-linking. Further, both modified and natural polysaccharides had the desired gelation time, good biocompatibility, and solubility in neutral solution. Novel methods such as 3D printing [164] and electrospun techniques [165] were investigated for fabricating wound dressing materials. Collagen, a natural structural protein that constitutes the extracellular matrix, is involved in different phases of wound healing events. Collagen can stimulate and recruit macrophages and fibroblasts, provide moisture or absorption, and create a microenvironment that hastens healing cascade by manipulating wound biochemistry. Oxidized regenerated cellulose in combination with collagen has the capability to bind with growth factors and inhibit matrix metalloproteinases in the wound site [166]. Collagen dressings can drastically decrease elastase levels in a wound environment, thereby disrupting the brutal cycle of chronic wounds and potentially promote wound healing.

A novel injectable hydrogel wound dressing was prepared from sericin, isolated from silk cocoon [167]. To improve the mechanical properties of sericin, it was cross-linked with sodium alginate using calcium ion as cross-linking agent to form an interpenetrating polymeric network. Further, silver nanoparticles were synthesized in situ by tyrosine moiety of sericin to enhance the antibacterial potential of the hybrid hydrogel. Injectable hydrogels are attractive as a minimally invasive non-surgical treatment for irregular wounds. CCK-8 assay showed the cytocompatible nature of sodium alginate/sericin and sodium alginate/sericin with silver nanoparticles. The bacterial growth and bacterial colony counting assay proved the antibacterial activity of sodium alginate/sericin with silver nanoparticles compared with the control. Wound contraction ratio determined after 12 days showed significant improvement with nanoparticles, demonstrating the potential of the hydrogel in wound healing.

Calcium alginate dressings are most promising in heavily exudative wounds where they enhance formation of granulation tissue. The alginate cross-link with sodium present in wounds could form a gel structure that provides an optimal moist healing environment while calcium stimulates cell migration, remodeling, and accelerates wound homeostasis [168]. In vivo studies in porcine and mouse models indicated that adipose-derived stem cells in gelatin wound dressings significantly accelerated the wound healing process and skin regeneration through enhanced cell growth and cell differentiation [169]. The histological examination showed that the wound dressing samples exhibited significantly lower inflammatory responses than that of the control sample and had hair follicle formation.

Amnioexcel^®^ and amniomatrix^®^ are cryopreserved amniotic suspensions developed by means of a proprietary CryoPrime™ process, which maintains the unique structural characteristics of amniotic allograft membrane. The product comprises collagen and alginate and has native tissue incorporating intact extracellular matrix and growth factors. It contributes structural tissues for repair, replacement, or reconstruction of damaged skin, especially in challenging and difficult to heal wounds.

## 6. Tissue Engineering

Tissue engineering or regenerative medicine is a rapidly evolving multidisciplinary field that refers to the practice of combining scaffolds, cells, and biologically active molecules for the recovery, maintenance, and improvement of tissue performance. Biomaterials contribute a pre-defined three-dimensional porous support structure within the anatomical site in which cells can attach, grow, and undergo spatial reorganization to generate new fully functional tissue. They also allow for the transfer of cells and desired biological factors to the targeted sites in the body. The fabrication of diverse geometrically shaped scaffolds with tunable pore sizes is particularly important for confinement of bioactives and cells for various biomedical applications [170]. Further, an interconnected porous structure is significant since the channels formed allow nutrients and signaling molecules to reach cultured cells [171].

An ideal biomaterial is expected to be biodegradable and bioresorbable to allow restoration of damaged tissue without inducing an inflammatory response. Thus, biomaterials provide adequate mechanical integrity for support during early tissue development, while in late development, it should have begun degradation itself such that it does not hinder additional tissue growth. A review describing the structure, synthesis, properties, fabrication method, and tissue engineering applications of hydrogels has been recently published [29].

Due to biodegradable and biocompatible characteristics, triblock polymers of PLA–poly(ethylene oxide)–PLA are employed as drug delivery carriers and tissue scaffolds [172]. Due to high porosity, PLA nanofibrous scaffolds are widely used for orthopedic, nerve, and soft tissue engineering applications [173]. The PVA-grafted lactide oligomers have demonstrated their potential application as a cartilage substitute in tissue engineering [174].

The most frequently used biological materials for scaffold fabrication are collagen, various proteoglycans, alginate-based substrates, and chitosan. In comparison to synthetic polymers, natural polymers are more biodegradable and bioactive, besides improving cell adhesion, proliferation, and growth. Nevertheless, fabricating homogenous and reproducible scaffold structures from fragile biological materials presents a challenge, for instance, in orthopedic applications. In order to avoid the problems using single-phase biomaterials, composite scaffolds comprising a number of biomaterials can be utilized.

The static microenvironment typically provided by hydrogels does not exactly simulate the complex heterogeneous and dynamic nature of the extracellular matrix. Recently, a number of novel fabrication technologies such as 3D bioprinting have emerged, which maintains the spatial heterogeneity essential for tissue integration, cellular activities, and biological processes [175]. The ability to create a printable and biocompatible cell-enriched hydrogel matrix is limited by the cytotoxic substances generated during cross-linking and photopolymerization reactions. An organ-on-chip system is considered as an emerging tool utilizing a microfluidic bioreactor device, where cells are microengineered to create tissue constructs which can reproduce organ-level functions [176]. The method is particularly useful for screening potential drug candidates and probing the fate of cells under different conditions, as it mimics in vivo conditions of the human body.

The application of novel approaches to concurrently control both the gelation process and the interactions between the gel and the extracellular matrix would widen the practicality of injectable hydrogels for drug delivery as well as in biomedical fields. Implanted hydrogels can either directly act as drug carriers or immobilize other drug delivery carriers at the targeted site. An injectable curcumin liposome-chitosan/β-glycerophosphate hydrogel (Cur.ps-H) system created through combined approaches involving a supercritical carbon dioxide technique and a thin-film evaporation method for tissue regeneration application has been reported [177]. This technique demonstrated maximum entrapment efficiency and stability when compared to thin film hydration. In vitro release studies of Cur.ps-H show sustained release of about 12 d, which suggest injectable liposome-hydrogels as a favorable delivery system for tissue restoration.

### 6.1. Polymers

#### 6.1.1. Collagen

Polymer scaffolds are utilized as space filling agents, as delivery vehicles for bioactives, and as three-dimensional structures as reservoirs of cells and growth factors for the formation of desired tissues. Collagen is used either alone or in combination with added constituents to promote biological and/or mechanical characteristics of the scaffold.

Investigations have led to the advancement of a collagen scaffold with optimized composition, cross-linking density, and pore size distribution for bone regeneration in vivo in minimally loaded calvarial defects. Improved mechanical properties with enhanced permeability for cell infiltration and vascularization have been demonstrated by introducing hydroxyapatite in a highly interconnected porous structure of collagen based on the two main constituents of bone. A biologically active composite scaffold was created either by adding a collagen slurry to a stable nano-hydroxyapatite suspension or immersing porous collagen scaffolds in nano-hydroxyapatite suspension after freeze-drying. The suspension method was found to be more reproducible, and the quantity of nano-hydroxyapatite loaded could be customized with greater ease than with the immersion technique [178]. It was reported that cell-free scaffolds prepared from collagen–glycosaminoglycan and collagen–calcium phosphate showed excellent healing compared to controls and tissue-engineered mesenchymal seeded constructs. The macrophage-led tissue remodeling response suggests that matrix deposited cell laden scaffolds adversely affect healing during bone regeneration [179].

The main hindrance in nerve regeneration is to develop an artificial neural graft that could imitate the extracellular matrix (ECM). It has been demonstrated in many investigations that electrospinning polymer blending is a simple and effective approach for fabricating novel biocomposite nanofibrous scaffolds. Data demonstrated that PLGA–silk fibroin–collagen scaffolds, particularly the one that contains 50% PLGA, 25% silk fibroin, and 25% collagen, is more suitable for nerve tissue engineering compared to PLGA nanofibrous scaffolds [180].

Results from cell regeneration and neurofilament protein expression analysis data indicated that the biocomposite nanofibrous, electrospun fabricated scaffold composed of poly(l-lactide-*co*-epsilon-caprolactone)/collagen I/collagen III behaves like ECM in nerves and therefore has a capability to be utilized a possible substrate for nerve regeneration [181]. It was demonstrated that aligned poly(3-hydroxybutyrate-*co*-3-hydroxyvalerate are better substrates for nerve tissue regeneration application [182]. The heparin/collagen constituting nerve growth factor layered onto the poly-l-lactide nanofibrous scaffolds also exhibited its potential role in peripheral nerve regeneration [183]. Thermosensitive hydrogels of PLGA-*g*-PEG constituting hyaluronic acid were researched for bone tissue engineering applications. It was indicated that the hydroxyapatite contents significantly enhanced the mechanical strength of hydrogels and the bioactivity of the composite.

The main limitations that restrict the extensive use of hydrogels in tissue engineering are uncontrollable swelling, toxic cross-linking processes, inadequate pseudoplastic rheological behavior, and lack of self-healing properties. An attempt has been made to circumvent these issues by modifying collagen hydrogel using 8-arm polyethylene glycol-maleimide cross-linker by thiol-Michael addition click reaction [184]. Novel self-healing, biodegradable, and biocompatible hydrogels demonstrated only minimum swelling (6%), as well as excellent shear thinning and cell compatibility properties.

#### 6.1.2. Alginates

Alginate-based hydrogels have been extensively evaluated in tissue engineering since they carry several characteristics akin to human extra cellular matrix. Recently, oxidized alginate-based hydrogels have drawn considerable attention as biodegradable materials for tissue engineering applications. Even with many promising advances, current technologies are limited in their capacity to regenerate cartilage bearing complex mechanical properties and biochemical composition. The regeneration of cartilage is investigated through a combination of chondrocytes or stem cells, stimulatory growth factors, and bioreactors enclosed within scaffolds preferably made from hydrogels. Development of cytocompatible adipocytes from human adipose stem cells entrapped in varying molecular weights of oxidized alginate hydrogel was reported [185]. In another study, a cell-enriched arginylglycylaspartic acid grafted oxidized alginate hydrogel used as a bioink for 3D-printing applications enhanced cell growth compared to unmodified alginate-based printed constructs [186]. Oxidized alginate/gelatin and oxidized alginate/fibrin hydrogels were investigated for bone tissue regeneration. A covalently cross-linked hydrogel made from galactosylated chitosan and oxidized alginate was tested as a scaffold for liver tissue engineering [187]. The oxidized alginate was also tested for cardiac repair, tissue fixation, and wound healing applications.

Creating a 3D vascularized architecture that supports cell viability due to enhanced oxygen permeation and nutrient transport continues to remain as a major hurdle in the field of bioengineering. A novel coaxial nozzle fabrication technology has been developed to print blood-vessel-like microchannels with mechanically stable shells containing cell-enriched structures within a calcium alginate gel matrix [188]. High cell viability attained by this method is mainly due to minimum exposure of the cell-encapsulated core to cross-linking agents while maintaining an effective cross-linking of external shell materials that provide an adequate mechanical stability to the formed structure. In another study, gelatin was incorporated to improve the mechanical stability of the structures along with hydroxyapatite and human mesenchymal cells for bone engineering. It was reported that Young’s modulus of the hydrogel systems was enhanced with the increase in hydroxyapatite content [189]. Calcium alginate microparticles loaded with bone morphogenic protein-2 (BMP-2) demonstrated improved osteogenic differentiation and controlled release of BMP-2 in goat multipotent stromal cells. Due to enhanced in vivo enzymatic degradation of gelatin, controlled release of BMP-2 was observed up to 4 weeks after subcutaneous implantation in mice [190]. AlgiMatrix^®^ is an example commercial hydrogel product typically used for 3D cell culture applications.

Recently, researchers have created a thermally actuating and mechanically strong construct prepared from Alginate/PNIPAAm ionic covalent entanglement hydrogels through extrusion-based 4D bioprinting [79]. The bioinks had a fixed alginate concentration of 4% (*w*/*v*) and varying NIPAAm concentrations (10%, 15%, or 20% *w*/*v*) with constant amounts of covalent cross-linking agent and UV initiator. By varying the amount of thermosensitive PNIPAAm network in the hydrogel, the gels demonstrated change in reversible length between 41–49% after heating and cooling between 60 °C and 20 °C (Figure 2). Blocked stresses noticed in tension were between 10–21 kPa. The opening and closing of smart valves in response to hot and cold water opens the possibility of such smart hydrogel materials to be used as self-assembling structures and various biomedical applications.

#### 6.1.3. Hyaluronic Acid

Hyaluronic acid offers many advantages as a scaffold including biodegradability, a primary intracellular component of connective tissues, creating an environment conducive for cell infiltration, and taking part in key cellular activities such as proliferation, tissue regeneration, and wound repair. Hyaluronic-acid-based polymers have been explored as cell carriers for bone, nerve, soft tissue, and smooth muscle engineering applications. Different chemical modifications on hyaluronic acid are performed to create new biocompatible, biodegradable mechanically strong scaffolding materials. The utilization of chitosan as a composite material for hyaluronic-acid-based hydrogel have been studied [191]. In vitro studies and implantation studies in osteochondral defects in rabbit knee joints with scaffolds encapsulated with chondrocytes showed ECM production effective for tissue repair. Similarly, implantation of tauroursodeoxycholic acid–PLGA microspheres in a hyaluronic acid hydrogel matrix in osteochondral defects in rats showed enhancement of tissue regeneration, defect filling, the generation of tissue structure, and calcification of the cartilage [192].

Development of an injectable in situ forming click cross-linked hyaluronic acid hydrogel loaded with chondrogenic differentiation factor, cytomodulin-2, and human periodontal ligament stem cells (hPLSCs) for musculoskeletal application has been described. The data observed signified that the hPLSC-loaded hydrogel exhibited greater and prolonged chondrogenic differentiation of hPLSCs [193]. Increased cell viability, biocompatibility, and better cell infiltration was noticed with the cross-linked hydrogel between poly (Nε-acryloyl-L-lysine) and hyaluronic acid [194]. The resultant composite hydrogels had excellent shape recovery properties after loading and unloading for 1.5 cycles (up to 90%) and displayed a highly porous microstructure. In vitro biocompatibility evaluation with pre-osteoblast MC3T3-E1 cells indicated that the pLysAAm/HA hydrogels could support cell viability and proliferation.

Evaluation of cellular activities and hyaluronidase expression displayed efficient proliferation, growth, and migration of the cells in heparin–hyaluronic acid hydrogels, when compared to hyaluronic-acid-only hydrogels [195]. It was disclosed that in this synergistic hydrogel, heparin chiefly acts as a binding domain for stem cells while hyaluronic acid mainly acts as a degradation site for human adipose tissue-derived mesenchymal-derived stem cell secreted enzymes.

Due to the viscoelasticity and fibrillar architecture, hyaluronic acid–collagen networks can be employed as materials that simulate the natural ECM, which could stimulate the differentiation of embedded stem cells [196]. Different natural polymers such as arginylglycylaspartic acid peptide-functionalized pectin, type I collagen, thiolated hyaluronic acid derivatives, and glycol chitosan have been used in combination with hyaluronic acid for cartilage tissue engineering [197].

In vivo studies of hyaluronic acid/chitosan in situ forming hydrogel demonstrated superior biocompatibility, rapid tissue regeneration, enhanced cell density, vascularization, and ECM formation [198]. Injectable hyaluronic acid–PEG hydrogel demonstrated its role in possible treatment for intervertebral disc degeneration to regain disc thickness and hydration [199]. These results are promising for tunable ECM-based materials for tissue engineering and regenerative medicine. A novel injectable, near-infrared irradiation-induced in situ forming reactive oxygen species cleavable hyaluronic acid hydrogel was used as a delivery vehicle for combined photodynamic-anticancer therapy with protoporphyrin and doxorubicin to obtain tunable on-demand drug release [200]. This versatile hydrogel possessing remarkable biocompatibility and biodegradability could act as a potential carrier for combined chemo-photodynamic cancer therapy in the near future.

## 7. Future Perspectives and Challenges

During the last few decades, many attempts have been made to develop targeted drug delivery systems that enable the drug delivery to specific sites, organs, tissues, cells, or organelles in the body to improve the therapeutic outcome. In this context, self-assembled nanocarriers to actively target overexpressed antigens or receptors in tumor cells are being explored greatly as an important approach to treatment. Hydrogel-based biosensors have been investigated for various biomedical applications [201] including cell metabolite and pathogen detection, tissue engineering, wound healing, and cancer monitoring, and identification of low-molecular-weight endogenous ligands such as glucose, lactate urea, and cholesterol. Recently, molecular imprinting techniques utilizing biological molecules with monomers and cross-linking agents have been used for fabricating biosensors. Fabrication of 3D-printed materials using hydrogels is receiving much attention these days [175]. The incorporation of nanotechnology and dynamic techniques such as 3D printing to the tissue engineering systems may narrow down the gaps currently existing among the in vitro models of tissue engineering. There is an emerging approach in hydrogel-based bioprinting for fabricating cell-laden scaffolds to develop anatomical size, complex tissue architecture, and tissue-specific functions [202].

## 8. Conclusions

Though research has been mainly directed towards macroscopic hydrogels, there is an ever-growing interest in micro and nanogels. Hydrogel nanoparticles are one of the most promising nanoparticulate drug delivery systems owing to their unique nature combining the features and characteristics of a hydrogel system with a nanoparticle. Polyelectrolyte nanogels can act as pharmaceutical carriers by accommodating oppositely charged low-molecular-weight drugs and macromolecules such as oligo- and polynucleotides (siRNA, DNA) as well as proteins as targeting motifs. The development of novel hydrogel formulations or improvement of existing hydrogels are presently drawing lot of attention from formulation and biomedical scientists, and have resulted in many commercial products.

## Figures and Tables

**Table 1:** Different techniques, materials, procedures, advantages, and disadvantages of hydrogels.

Techniques	Types of Materials Used	Procedure	Advantages	Disadvantages	Reference
Hydrophobic interaction	Hydrophilic monomers and hydrophobic comonomers	Free radical copolymerization of a hydrophilic monomer with a hydrophobic co-monomer	Absence of cross-linking agents and relative ease of production	Poor mechanical characteristics	[3]
Ionic interaction	Polyelectrolyte solution and multivalent ions of opposite charge	Ionic interaction through simple ion exchange mechanisms and complex formation	Cross-linking takes place at room temperature and physiological pH Properties can be fine-tuned by cationic and anionic constituents	Limited to ionic polymers and sensitive to impurities	[4]
Hydrogen bond	Polymeric functional groups of high electron density with electron-deficient hydrogen atom	Self-assemble through secondary molecular interactions	Increase in polymer concentration can increase the stability of gel.	Influx of water can disperse/dissolve the gel within short duration.	[5]
Bulk polymerization	Monomers and monomer-soluble initiators	The polymerization reaction is initiated with radiation, ultraviolet, or chemical catalysts at low rate of conversions	A simple and versatile technique to prepare hydrogels with desired physical properties and forms	Increase in viscosity during high rate of polymerization reaction can generate heat Weak polymer structure	[6]
Solution polymerization/	Ionic or neutral monomers with the multifunctional cross-linking agent	Reaction initiated thermally with UV irradiation or by redox initiator system	Control of temperature Performed in non-toxic aqueous medium at room temperature High polymerization rate	To be washed to eliminate reactants, the polymers, and other impurities	[7]
Suspension polymerization	Hydrophilic monomers, initiators, cross-linkers, and suspending agent	The monomers and initiator are dispersed in the organic phase as a homogenous mixture	Directly usable as powders, beads, or microspheres	Restricted to water insoluble polymer Cooling jacket required to dissipate heat Requirement of agitators and dispersant	[2]
Grafting	Viny polymers, initiators and cross-linking agents	Covalent bonding of monomers on free radicals generated on stronger support structures	Improve functional properties of the polymer	Difficulty of characterizing side chains	[8]
Irradiation	High energy gamma beams and electron beams as initiators	Irradiation of aqueous polymer solution results in the formation of radicals and macroradicals on the polymer chains	Pure, sterile, residue-free hydrogel Does not require catalyst and other additives Irradiation dose can control swelling capacity	Irradiation can cause polymer degradation via chain scission and cross-linking events	[9]
Step growth polymerization	Bi or multifunctional monomers and each with attest two sites for bonding	Multifunctional monomers react to form oligomers resulting in long chain polymers	No initiator is required to start the polymerization and termination reactions	Prolonged reaction times required to achieve a high degree of conversion and high molecular weights	[10]

**Table 2:** Different kinds of stimuli responsive hydrogels.

Types	Description	Examples	References
Temperature responsive	Change in temperature disturbs the equilibrium exists between hydrophilic and hydrophobic segments of the polymer chain and induce the sol-gel transformation	Pluronic, poloxamer, poly(acrylic acid), poly(*N*-isopropylacrylamide), and tetronic	[11]
pH responsive	Change in pH results in swelling/deswelling behavior due to the changes in hydrophobicity of the polymer chain	Chitosan, guar gum succinate, kappa-carrageenan, poly (ethylene imine), poly(acrylamide), poly(acrylic acid), poly(diethylaminoethyl methacrylate), poly(dimethylaminoethyl methacrylate), poly(ethylacrylic acid), poly(hydroxyethyl methacrylate), poly(methacrylic acid), poly(propylacrylic acid), and poly(vinyl alcohol)	[12]
Photoresponsive	External stimulus of either visible or UV light initiates sol-gel transformation	Azo benzene-poly(2-hydroxyethyl methacrylate), azo benzene-bovine albumin, triphenylmethane leuco derivatives, and trisodium salt of copper chlorophyllin-poly(*N*-isopropylacrylamide)	[13]
Electroresponsive	Upon the application of an electric field, deswelling or bending takes place, based on the shape and position of the gel relative to the electrodes	Agarose, calcium alginate, carbomer, chondroitin sulphate, hyaluronic acid, partially hydrolyzed polyacrylamide, polydimethylaminopropyl acrylamide, and xanthan gum	[14]
Ultrasonically responsive	External application of ultrasonic waves modulates the drug release from the hydrogel matrix	Ethylene vinyl acetate, poly (2-hydroxyethyl methacrylate), poly(bis(p-carboxyphenoxy)alkane-anhydrides, poly(lactide-*co*-glycolide, polyglycolide and polylactide	[15]
Magneto responsive	Application of heating, mechanical deformation, or external magnetic field to magnetic nanoparticles, such as nanoparticles of magnetite, maghemite, and ferrite	Alginate-xanthan cross-linked with Ca2+ magnetic nanoparticles, hemicellulose hydrogels with magnetic iron oxide (Fe_3_O_4_), methacrylated chondroitin sulfate with magnetic nanoparticles, poly(*N*-isopropylacrylamide), and xanthan-bovine serum albumin-magnetic nanoparticles	[16]
Glucose responsive	Hydrogel as a self-regulated, insulin-delivery system discretely switching release at normoglycemia	Catalase, insulin, phenylborate derivative {4-(1,6-dioxo-2,5-diaza-7-oxamyl) phenylboronic acid in combination with poly(*N*- isopropylmethacrylamide), and poly(2-hydroxyethyl methacrylate-*co*-*N,N*-dimethylaminoethyl methacrylate) in combination with glucose oxidase	[17]
Ionic strength	Varying ionic strength and pH can expand the polyelectrolytes resulting in dissociation of ionizable groups and subsequent drug release	Alginic acid, carboxymethyl cellulose, carboxymethyl starch, carrageenan, cellulose sulfate, chitosan, dextran sulfate, eudragit E, RL, RS, and hyaluronic acid	[18]
Inflammation responsive	pH changes at the inflammatory site resulted in drug release or pH-responsive hydrogel with inflammatory responsive characteristics and the capability to passively targeting macrophages	Aliphatic polyketals and pH-responsive polymers	[19]

**Table 3:** Selected examples of hydrogel-based commercial dosage forms for oral delivery.

Commercial Product	Polymers	Active Constituents	Dosage Form	Application	Manufacturer
Buccastem^®^ M	Povidone K30, xanthan gum, locust bean gum	Prochlorperazine maleate	Tablet	Nausea and vomiting in migraine	Alliance
Biotene	Carbomer and hydroxyethyl cellulose	Nil	Gel	Oral moisturizing agent in dry mouth	GlaxoSmithKline
Gengigel^®^	Hyaluronan	Nil	Gel	Mouth and gum care-oral ulcers	Oral Science
Hydrogel 15%	Carbomer in ozonized sunflower oil	Ozone	Gel	Oral health	Honest 03
Lubrajel™ BA	Glyceryl acrylate and glyceryl polyacrylate	Nil	Gel	Oral moisturizing agent	Prospector
Nicorette^®^	Hydroxypropyl methylcellulose	Nicotine	Chewing gum	Smoking cessation	GlaxoSmithKline
Nicotinell^®^	Xanthan gum and gelatin	Nicotine	Chewing gum	Smoking cessation	GlaxoSmithKline
Zilactin-B Gel^®^	Hydroxypropyl cellulose	Benzocaine	Gel	Local anesthetic in minor oral problems	Blairex laboratories Inc.
ZuplenzTM	Polyethylene glycol 1000, polyvinyl alcohol and rice starch	Ondansetron	Soluble oral film	Chemotherapy, radiation, surgery-induced nausea and vomiting	Aquestive Therapeutics

**Table 4:** Selected commercial examples of hydrogel-based ocular drug delivery systems and soft contact lens materials.

Product Name	Principal Components	Indications	Manufacturer
Biofinity^®^	Silicone hydrogel	Continuous wear up to 7 days. Corrects near sightedness and farsightedness	Cooper vision
Air optix^®^ night and day^®^aqua	Lotrafilcon-A (fluoro-silicone hydrogel)	Continuous wear up to 7 days. Corrects near sightedness and farsightedness	Alcon
Retisert^®^	Fluocinolone acetonide, silicone elastomer and polyvinyl alcohol membrane	Deliver long term control of inflammation	Bausch and Lomb
Lacrisert^®^	Hydroxypropyl cellulose	Moderate to severe dry eyes	Bausch and Lomb
Systane^®^	Propylene glycol	For use as a lubricant to prevent further irritation or to relieve dryness of the eye	Alcon
Restasis^®^	Cyclosporine, carbomer copolymer Type A	Indicated to increase tear production	Allergan
Proclear (Omafilcon B)	2-hydroxy-ethylmethacrylate and 2-methacryloxyethyl phosphorylcholine cross-linked with ethylene glycol dimethacrylate	Indicated for daily wear for the correction of visual acuity	Cooper vision
Clintas Hydrate^®^	Carbomer	Lubricating eye gel for occasional dry eye discomfort	Altacor
Dailies^®^ AquaComfort	Nelfilcon A polymer (polyvinyl alcohol partially acetalized with *N*-formylmethyl acrylamide)	Optical correction of refractive ametropia	Ciba vision
Systane^®^ gel drops	Polyethylene glycol 400, propylene glycol	For the temporary relief of burning and irritation due to dryness of the eye	Alcon
Hylo^®^ gel	Sodium hyaluronate, citrate buffer, sorbitol	Long lasting dry eye relief	Candorvision
Iluvien^®^	Fluocinolone acetonide, polyvinyl alcohol, and silicone adhesives	Treatment of diabetic macular edema	Alimera Sciences
Yutiq™	Fluocinolone acetonide, polyvinyl alcohol	Treatment of chronic non-infectious uveitis affecting the posterior segment of the eye	EyePoint Pharmaceuticals Inc.
Ozurdex^®^	Dexamethasone, poly (d,l-lactide-*co*-glycolide)	Macular edema, non-infectious uveitis	Allergan

**Table 5:** Selected commercial examples of hydrogel-based wound dressing materials.

Product Name	Principal Components	Indications	Manufacturer
Helix3-cm^®^	Type 1 native bovine collagen	Management of burns, sores, blisters, ulcers, and other wounds	Amerx Health Care Corp.
3M™ Tegaderm™ hydrogel wound filler	Hydrocolloid dressing	Low to moderate draining wounds, partial- and full-thickness dermal ulcers	3M Health Care Ltd.
AquaSite ^®^amorphous hydrogel dressing	Glycerin based hydrocolloid dressing	Provide moist heat healing environment and autolytic debridement	Integra Life Science Corp.
Algicell^®^ Ag calcium Alginate dressing with antimicrobial silver	Calcium alginate ionic silver	Effective against a broad range of bacteria and more absorption of drainage	Integra Life Science Corp.
INTRASITE^®^ gel hydrogel wound dressing	Modified carboxymethyl cellulose, propylene glycol	Re-hydrates necrotic tissue, facilitating autolytic debridement minor burns, superficial lacerations, cuts, and abrasions	Smith &Nephew Healthcare Limited
Microcyn^®^ skin and wound hydrogel	Hypochlorous acid	All types of chronic and acute wounds as all types of burns	Microsafe group
Prontosan^®^ wound gel	Polyhexanide and betaine	Cleansing and moisturizing of skin wounds and burns	Bbraun
Purilon^®^ gel, Regenecare^®^ wound gel	2% lidocaine collagen, aloe and sodium alginate	Pressure ulcers, cuts, burns, and abrasions	MPM Medical
Cutimed^®^ gel	Carbomer 940	Supports autolytic debridement in necrotic and sloughy wounds	BSN Medical
Viniferamine^®^ wound hydrogel Ag	Glycerin Metallic silver	Partial and full thickness wounds with signs of infection and little to no exudate	McKesson
HemCon^®^ bandage PRO	Chitosan	Providing hemostasis, antibacterial barrier against wide range of microorganisms	TriCol biomedical Inc.
Hyalofill^®^-F and R	Hyaluronic acid in fleece and rope	Absorbs wound exudate, promotes granulation tissue formulation, supports healing process	Anika
CMC fiber dressing	Carboxymethyl cellulose	Absorptive dressing for moderate to heavy exudate	Gentell
Inadine™ (PVP-1) non-adherent dressing	Polyethylene glycol, povidone iodine	Ulcers deriving from different etiologies, chronic wounds	3M Health Care Ltd.

## Data Availability

The data presented in this study is contained within this article.
